# Impact of thyroid hormone treatment on maternal pregnancy outcomes in women with subclinical hypothyroidism without TPOAb: a retrospective cross-sectional study

**DOI:** 10.1186/s13044-023-00171-7

**Published:** 2023-09-11

**Authors:** Georgiana Sitoris, Flora Veltri, Emna Jelloul, Pierre Kleynen, Serge Rozenberg, Kris G. Poppe

**Affiliations:** 1 Endocrine Unit Centre Hospitalier Universitaire Saint-Pierre, Université Libre de Bruxelles (ULB), Rue Haute 322, Brussels, 1000 Belgium; 2 Departement of Gynecology and Obstetrics, Centre Hospitalier Universitaire Saint-Pierre, Université Libre de Bruxelles (ULB), Rue Haute 322, Brussels, 1000 Belgium

**Keywords:** Pregnancy, Thyroid hormone treatment, Maternal outcomes, Subclinical hypothyroidism

## Abstract

**Background:**

Evidence on the impact of thyroid hormone treatment (LT4) on maternal pregnancy outcomes in women with subclinical hypothyroidism (SCH) without thyroid peroxidase antibodies (TPOAb) positivity is scarce.

**Methods:**

Single centre, cross-sectional study in 1460 women screened for TSH, free T4 and TPOAb at median 13 (11–17) weeks of gestation during the period 2013–2014. Exclusion criteria were twin- and assisted reproduction pregnancies, TPO positivity, overt thyroid dysfunction, and treatment with LT4 before screening. The impact of LT4 on maternal pregnancy outcomes was investigated in a group of 53 women with SCH (TSH > 3.74 mIU/L) in which LT4 was initiated at median 13 (10–22) weeks (treated group). The control group included 18 women with SCH (TSH > 3.74 mIU/L). The prevalence of pregnancy complications in these two groups was compared with that in a euthyroid reference (REF) group of 1389 women (TSH ≤ 3.74 mIU/L).

**Results:**

The prevalence of pre-eclampsia and gestational diabetes (GDM) was higher in the control group vs the REF group (16.7% vs 5.0% and 27.8% vs 18.9%; *p* = 0.017 and *p* = 0.016, respectively), but comparable in the treated group vs the REF group (7.6% vs 5.0% and 22.6% vs 18.9%; *p* = 0.918 and 0.676, respectively). The prevalence of iron-deficiency anaemia was lower in the treated vs the REF group (17.0% vs 32.5%; *p* = 0.017).

**Conclusion:**

Pregnant women with untreated SCH and without TPOAb positivity had a higher prevalence of pre-eclampsia and GDM compared with euthyroid women, while this was not the case in women with treated SCH, even when it was initiated after the first trimester.

## Introduction

In the guidelines on the management of thyroid disorders in pregnancy, it is proposed to treat women with LT4 according to the severity of the subclinical hypothyroidism (SCH) [1]. The recommendation is the strongest in case of overt hypothyroidism, followed by women with SCH and thyroid autoimmunity (TAI) and women with TAI and TSH levels > 2.5 mIU/L < upper limit of the local TSH reference range [1, 2]. Soon, guidelines will be revised, based on recent studies, that do not show a beneficial impact of LT4 on life birth and recurrent miscarriage rates in euthyroid women with TAI [3, 4]. In women with SCH and no TAI, the TSH thresholds to initiate LT4 are 4.0 mIU/L (weak recommendation) and 10.0 mIU/L (strong recommendation), respectively; both based on the study by Nazarpour et al. [1, 2, 5]. In that study, 254 pregnant women with SCH (TSH 4.0–10.0 mU/L) but without TAI were randomized to either receive or not LT4 [5]. The preterm birth (PTB) rate was compared to that in 940 euthyroid pregnant women without TAI. In the LT4 treated group, the PTB rate decreased to 7.3% compared to 19% in the control group (*p* = 0.04). Since this is the only study in pregnant women with SCH and no TAI, the American College of Obstetricians and Gynecologists (ACOG) recommends no LT4 for these women, but only in case of overt hypothyroidism [6].

Moreover, evidence on the impact of SCH (with a serum TSH level > 4.0 mIU/L) on pregnancy outcomes is limited and the results are conflicting [5, 7, 8]. Only recently, two studies in pregnant women with SCH and no TAI were published, showing a higher prevalence of gestational diabetes (GDM), gestational anaemia, pre-eclampsia, and foetuses small for gestational age compared with euthyroid women [9, 10].

The aim of this study was to investigate the prevalence of pregnancy complications (pre-eclampsia (PE), GDM, iron deficiency anaemia (IDA), blood loss at birth, emergency caesarean section (C-section), PTB and birth weight) in two groups of women with SCH without TPOAb positivity. On one hand, a control group not treated with LT4, and on the other hand women treated with LT4. The prevalence of the pregnancy outcomes in these two groups was then compared with that in a euthyroid reference (REF) group also without TAI.

## Material and methods

### Overall study design / definitions

The obstetric clinic is part of the downtown public university hospital CHU Saint-Pierre in Brussels, Belgium. In this cross-sectional retrospective study (period between 2 Jan 2013 to 31 Dec 2014), we included women with ongoing pregnancies, who performed their entire biological work-up and obstetric follow-up in our centre throughout pregnancy. During the first antenatal consultation, demographic parameters and obstetric data with a biological analysis including serum TSH, FT4, thyroid peroxidase antibodies (TPOAb), and ferritin are systematically collected. Later in pregnancy, an oral glucose tolerance test (OGTT) was performed in all pregnant women (except for women with fasting glycemia levels ≥ 92 mg/dl, who were not included in the study).

Exclusion criteria for this study were women with known diabetes mellitus and thyroid disorders before pregnancy, multiple and assisted pregnancies, thyroid disorders (either or not treated with antithyroid drugs or LT4 in case of TSH levels ≤ 3.74 mIU/L) before or after screening, and TPOAb positivity.

**Fig. 1:**
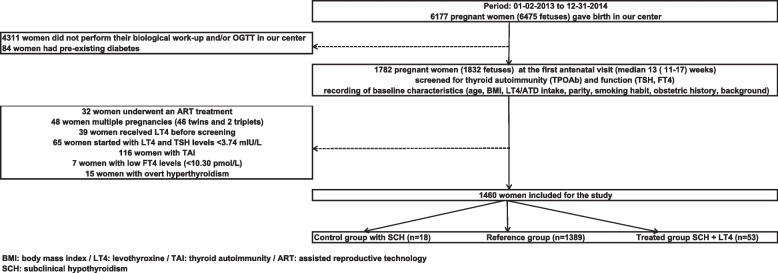
Flowchart of the study selection process

In Fig. 1, we illustrate the study selection process in more detail.

In a previous study, we determined institutional trimester-specific limits for serum TSH and fT4, according to the ATA-GL recommendations [1]. Our reference range for serum TSH (2.5–97.5^th^ percentile) is 0.06–3.74 mIU/L, and 10.29–18.02 pmol/L for serum FT4 [11]. TAI was defined as TPOAb levels ≥ 60 kIU/L. For this study, three groups were established based on these TSH levels; two groups of women with SCH, one treated group (*n* = 53) and one not treated (control group; *n* = 18). For comparison of pregnancy complications, euthyroid women with TSH levels  ≤ 3.74 mIU/L (*n* = 1389) served as the REF group.

LT4 treatment was initiated by the gynaecologist and/or the endocrinologist according to the ATA-GL of 2011 (from TSH levels > 2.5 mIU/L on, independently of TAI) [12]. LT4 dosage was adapted when TSH levels at control were not < 2.5 mIU/L. The reasons why some women with a treatment indication (the untreated SCH group) did not receive LT4, could have been multiple (treatment refusal, not proposed by the physician, etc.…) but are not always identifiable in the files.

Gestational age was based on ultrasound findings and expressed in full weeks and days of amenorrhea. Smoking was stratified as yes/no (women who stopped smoking during pregnancy were also considered as smokers).

Outcome measures: during all prenatal consultations, a standard urine test strip for proteinuria was performed and if after 20 weeks of pregnancy women developed hypertension and/or a clinical suspicion of pre-eclampsia (PE), then a quantitative proteinuria measurement was done. PE was defined as a systolic blood pressure ≥ 140 mmHg and/or a diastolic blood pressure ≥ 90 mmHg, associated with a proteinuria > 0.3 g/24 h after 20 weeks of amenorrhea.

GDM was diagnosed after administration of 75 g glucose during an OGTT performed between 24–28 weeks of pregnancy when fasting glucose ≥ 92 mg/dL or 1-h postprandial glycaemia ≥ 180 mg/dL or 2 h ≥ 153 mg/dL) [13].

IDA was present when haemoglobin levels are < 12, 11 or 10.5 g/dl during the first, second or third trimester, respectively, together with a serum ferritin < 15 ug/L. Significant postpartum haemorrhage was considered as ≥ 500 mL, and only considered after spontaneous birth. PTB was defined as birth < 37 weeks.

### Serum assay

All provisions were implemented by the laboratory of hormonology of our institution.

Serum TPO-Ab levels were measured using the Elecsys electrochemiluminescence immunoassays on a Cobas 6000 immunoanalyzer (Roche Diagnostics, Mannheim, Germany). The inter- and intra-assay CV for TPO-Ab is ≤ 5% and ≤ 7%, respectively; reference values are < 34 kIU/L. Serum thyrotropin was measured using a sandwich immunoassay with electrochemiluminescence detection on a Roche analytical platform (TSH, Roche Diagnostics, Mannheim, Germany). The coefficient of variation (intermediate precision) is 3.5% at 1 mIU/L; reference values: 0.27–4.2 mIU/L. Serum free T4 (FT4) was measured using a competitive immunoassay with electrochemiluminescence detection on a Roche analytical platform (FT4, Roche Diagnostics). The coefficient of variation (intermediate precision) is 3.5% at 12.8 pmol/L; reference values: 12–22 pmol/L. Serum Ferritin levels were measured using the Chemiluminescence Centaur XP Siemens immunoanalyzer; reference values 15–300 ug/L and total imprecision CV 3.7%. Plasma glucose was measured by an automated colorimetric-enzymatic method on a Hitachi/ Roche-Modular P analyser; CV is 1%.

### Statistical analysis

Data were stored in a Microsoft Excel database and statistical analyses performed using StatPlus:mac, Analyst Soft Inc.—version v8.

Descriptive statistics are presented as mean ± standard deviation (SD) for normally distributed and median (interquartile range (IQR)) for skewed variables.

Comparison between the control and treated group versus the REF group was done by Chi^2^ or Fisher’s exact tests (according to the number of events) for categorical data and by a T-test or Mann–Whitney U test for continuous data (according to the distribution).

*P* values for differences in the prevalence of pregnancy outcomes between groups are given as crude and adjusted ones, after correction for age (> 34 years), BMI (≥ 30 kg/m^2^), pre-existing hypertension, GDM, and pre-eclampsia, depending on the outcome investigated (cf details in the legend of Table 3).

Statistical tests were considered significant whenever *p* < 0.05/2 = 0.025 for multiple (two) comparisons.

## Results

Table 1 shows baseline demographic and obstetric parameters in the study groups.

**Table 1:**
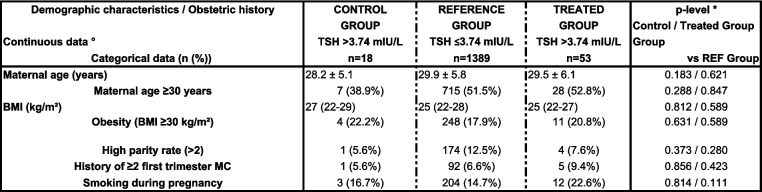
Baseline demographic and obstetric parameters in the study groups

°Continuous data are expressed as mean ± SD or as median (interquartile range; IQR) according to the distribution

*BMI* Body mass index, *MC* Miscarriage

*p-value of 0.025 (*p*<0.05/2) was considered as significant, taking two comparisons into account

Mean ± SD maternal age in the SCH control group (28.2 ± 5.1 years) and treated group (29.5 ± 6.1 years) was comparable with that in the REF group (29.9 ± 5.8 years); *p* = 0.183 and 0.621, respectively. The prevalence of obesity in the control group (22.2%) and treated group (20.8%) was comparable with that in the REF group (17.9%); *p* = 0.631 and 0.589, respectively. High parity rate, history of $\ge$ 2 miscarriages, and tobacco use were all comparable between groups.

Table 2 shows thyroid parameters and treatment details in the study groups.

**Table 2:**
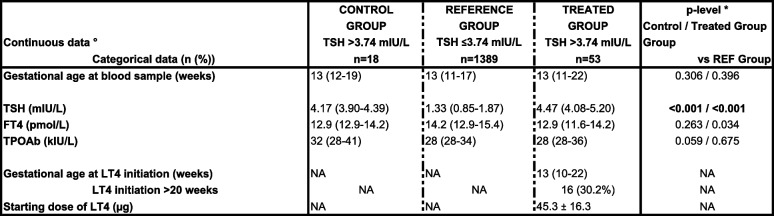
Thyroid parameters and treatment details in the study groups

°Continuous data are expressed as mean ± SD or as median (interquartile range; IQR) according to the distribution

*TSH* thyrotropin, *FT4* free thyroxine, *TPOAb* thyroid peroxidase autoantibodies, *LT4* levothyroxine, *NA* not applicable

*p-value of 0.025 (*p*<0.05/2) was considered as significant, taking two comparisons into account

Median (IQR range) gestational age at blood sample in the control group (13 (12–19) weeks) and treated group (13 (11–22) weeks) was comparable with that in the REF group (13 (11–17) weeks); *p* = 0.306 and 0.396, respectively.

Median (IQR range) serum TSH levels in the control group (4.17 (3.90–4.39) mIU/L) and treated group (4.47 (4.08–5.20) mIU/L) were, by default, higher compared with those in the REF group (1.33 (0.85–1.87) mIU/L); both *p* < 0.001, respectively. Median (IQR range) FT4 levels in the control group (12.9 (12.9–14.2) pmol/L) and treated group (12.9 (11.6–14.2) pmol//L) were comparable and lower compared with those in the REF group (14.2 (12.9–15.4) pmol/L); *p* = 0.263 and 0.034, respectively. The median (IQR range) gestational age at which LT4 was started was 13 (10–22) weeks, of whom 30.2% started treatment after 20 weeks of gestation. The mean starting dose of LT4 was 45.3 ± 16.3 ug / day, and in eleven women (20.8%), the dose had to be adapted in a mean 7 weeks after the initiation of LT4.

Table 3 shows the number (%) of maternal pregnancy complications in the study groups.

**Table 3:**
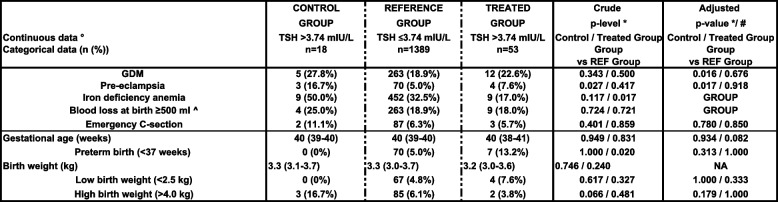
Maternal pregnancy complications data in the study groups

°Continuous data are expressed as mean ± SD or as median (interquartile range; IQR) according to the distribution

*GDM* gestational diabetes mellitus, *LT4* levothyroxine, *^* calculated for natural births

*p-value of 0.025 (*p*<0.05/2) was considered as significant, taking two comparisons into account

*/# *p* values adjusted

for pre-eclampsia taking into account pre-existing hypertension, obesity and age >34 years

for GDM taking into account obesity and age >34 years

for C-section taking into account obesity

for preterm birth taking into account pre-eclampsia, obesity, GDM and intrauterine growth restriction

for low birth weight taking into account preterm birth and BMI <18 kg/m^2^

for high birth weight taking into account GDM, obesity and age >34 years

*NA* not applicable

The prevalence of GDM was higher in the control vs REF group (27.8% vs 18.9%; *p* = 0.016) and comparable between the treated and REF group (22.6% vs 18.9%; *p* = 0.676).

The prevalence of PE was higher in the control vs REF group (16.7% vs 5.0%; *p* = 0.017) and comparable between the treated and REF group (7.6% vs 5.0%; *p* = 0.918).

The prevalence of IDA was comparable between the control and REF group (50.0% vs 32.5%; *p* = 0.117) and lower in the treated vs REF group (17.0% vs 32.5%; *p* = 0.017. The prevalence of other outcomes (preterm birth, birth weight, blood loss at birth and emergency C-section) was comparable between groups.

## Discussion

To the best of our knowledge, this is the first study in pregnant women with SCH and no TPOAb positivity showing a beneficial impact of LT4 treatment on several maternal pregnancy outcomes such as PE, GDM and iron deficiency anaemia. In the current guidelines on thyroid and pregnancy, there is a weak recommendation to treat this group of pregnant women, since only one interventional study has been performed showing a decreased PTB rate in LT4 treated women with TSH levels > 4.0 mIU/L [1, 5]. Furthermore, observational data on the impact of SCH without TAI on pregnancy outcomes are scarce. In original studies [7, 14], a meta-analysis [15] and in an individual-participant data meta-analysis [8], SCH has been associated with more foetal and maternal complications like miscarriage, pregnancy related hypertension, PE, placental abruption, and PTB. However, it is not always clear if the impact of SCH was corrected for the presence of TAI. Another reason why few studies are published in pregnant women with SCH without TPOAb is because the latter is the most frequent cause of SCH [16]. Recently, two Chinese studies including > 200 pregnant women without TAI and an Italian study with TSH levels 4.0–10.0 mIU/L showed a higher prevalence of GDM, PE and PTB [9, 10, 17]. In analogy with the study by Zhu et al., we observed a higher prevalence of GDM in women with SCH [9].

In a meta-analysis, it was shown that in studies applying a TSH cut-off > 4.0 mIU/L an increased odds for GDM, regardless of TAI status was present (OR 1.60; 95% CI: 1.33–1.93) [18]. During pregnancy, thyroid hormone (TH) has dual effects on islet cells via the sensitivity and its degradation. The combination of SCH and pregnancy can lead to the development of GDM, mediated via low(er) serum hCG and FT4 levels [19–22]. The prevalence of GDM in our treated SCH group was comparable, with that in the euthyroid REF group (but not lower). Mechanisms explaining how LT4 can impact on GDM during pregnancy may include an improved insulin sensitivity and/or by limiting weight increase. Noteworthy is that we did not document a history of previous GDM episodes and did not measure TgAb, the latter have also been associated with the presence of GDM independently of TPOAb [20].

Concerning the association with PE, our results are in line with those reported by Li et al. and Magri et al., but also with a recent meta-analysis in which a higher (but also a lower) TSH level was associated with a higher risk of PE (*p* < 0.001) [10, 17, 23]. Some studies reported no association between SCH and PE, but that might have been due to other definitions of SCH and the fact that no corrections were made for obesity, age, and pre-existing maternal hypertension [24, 25]. The link between thyroid dysfunction and PE has been described by Wilson et al. and allude to the fact that TH can have cardiovascular effects (genomic and nongenomic) and lead to endothelial dysfunction [26]. More recently, a mediating role of serum hCG levels on FT4 levels has been proposed, that might explain the association between PE and both hypo- and hyperthyroidism [27]. In our study, treated women had a prevalence of PE that was comparable with that in the REF group.

Concerning PTB, we had too few cases in the control group to perform a statistical analysis. Moreover, in a meta-analysis, the association between SCH and PTB was no longer significant after additional adjustment for TPOAb positivity either [8]. In literature, PTB is present in 5% to 15% of births and is an important direct cause of morbidity and mortality in young children, and a risk factor for other diseases later in life [28]. TH regulate placental development and function, foetal growth, and neuropeptides involved in the onset of labour [29–31]. In the only interventional study in literature, PTB decreased from 19% in the control group to 7.3% in the treated group (RR: 0.38; 95% CI: 0.15–0.98; *p* = 0.04) [5]. Concerning the impact of LT4 on PTB, we noted no impact after adjustment for confounders.

Iron deficiency anaemia tended to be more frequent in the control group versus the REF group (50% vs 32%). In the meta-analysis by Yang et al., no association between SCH and gestational anaemia was present (OR 1.55, 95% CI: 0.99– 2.44), although it should be noted that the index of heterogeneity was high (I^2^ of 83%), and that for the definition of SCH old (2.5 mIU/L) and new (4.0 mIU/L) cut-off values for TSH were used [32]. In the study by Zhu et al., the risk of gestational anaemia was significantly higher in women with TSH levels > 4.0 mIU/L (aOR 3.28 (95% CI: 1.60–6.75)) [9]. The underlying mechanisms explaining the association between anaemia and thyroid function remain unclear; a few studies have shown that iron deficiency might affect thyroid function due to an impaired efficacy of TPO activity as well as the conversion from T4 to T3 or on the Krüppel-like factor 9, an essential factor for erythroid maturation and T lymphopoiesis [33, 34]. Gestational anaemia and iron deficiency is of main importance for a normal pregnancy outcome, and not always considered in the studies associating thyroid disorders with pregnancy outcomes [35, 36]. In our study, in treated women, the prevalence of IDA was significantly lower compared with that in the REF group. An explanation could be that hepcidin overexpression leads to iron-restricted anaemia. In one study in patients with Hashimoto’s disease, the median level of hepcidin was significantly lower after LT4 treatment (7.7 (6.2–13.0) vs 17.4 (7.6–20.4) ng/mL; p = 0.002) [37]. Furthermore, TH play an important role in haematopoiesis by regulating gene expression and EPO secretion in kidneys, which stimulates proliferation of erythrocyte precursors. In one study, impaired erythropoiesis in case of hypothyroidism was reversible following restoration of euthyroidism [36, 38].

Blood loss at birth, emergency C-section and birth weight were not different between groups.

Our study results might add (some) evidence in favour of recommending LT4 treatment for pregnant women with SCH (TSH between 4.0–10.0 mIU/L), irrespective of the TPOAb status. In a recent editorial, Stagnaro-Green favours LT4 treatment based on a study on the effects of LT4 on the intellectual development of the offspring of mothers with SCH and no TPOAb [39, 40]. However, Pearce et al. provide arguments against a systematic treatment such as the fact that the cited study was not an RCT and that the other evidence on the impact of LT4 is still limited to the study by Nazarpour et al. on PTB [5, 41].

Another important observation in our study was the fact that the initiation of LT4 during late first to early second trimester, was also associated with a beneficial impact on outcomes such as PE and GDM that typically occurs later in pregnancy. This is in contrast with cognitive endpoints as outcome, in which LT4 probably needs to be given very early during pregnancy [42, 43].

Limitations of our study should be acknowledged. It is a retrospective analysis, and therefore, it was not always clear why some women were treated and others not. Two thirds of women in the control group had a second TSH measurement, in a mean 10 weeks later than the initial one (25 vs 15 weeks), and therefore, they cannot be used to confirm the initial diagnosis of SCH (data not shown). However, it highlights a TSH control might have its importance, but should be done within the same gestational period (although ATA-GL does not propose this).

Due to the relatively small number of women that were treated with LT4, we were unable to adjust for the starting period of LT4 (before or after 20 weeks of pregnancy).

The diagnosis of TAI could have been improved by adding TgAb measurement, and performing an ultrasound, that has a high positive predictive value (88%) [44, 45]. Furthermore, was the first blood sample performed outside the first trimester in a substantial number of women, what might have led to a lower diagnostic rate of TAI, due to the physiological change in the Th1 to Th2 phenotype, mediated by higher progesterone levels [46]. In the paper by Ekinci et al., it was shown that the odds of having positive TPOAb was lower at the second trimester of pregnancy (OR 0.04; 95% CI: 0.02–0.80; *p* = 0.03) [47]. Concerning GDM as outcome, we included only women that performed an OGTT, and thus some women with a fasting increased glycaemia in the early pregnancy were therefore not included, introducing a potential bias. For some outcomes (PTB), we were able to analyse only small numbers, what hampered us to draw firm conclusion on that point. Our data were not corrected for the iodine status. However, from a previous study performed in pregnant women in Brussels, we know there is a moderate deficiency, but without thyroid dysfunction [48].

Strengths of the study are the multiple outcomes investigated, the real-world setting and the fact that we were able to include a SCH control and euthyroid reference group for comparison with women with SCH who received LT4.

Since our results might be in favour of treating women with SCH, it might add some evidence in favour of a systematic screening for serum TSH in pregnant women [49].

## Conclusions

In this real-world retrospective study, women with SCH and no TPOAb positivity might have had a higher prevalence of gestational diabetes and preeclampsia compared with euthyroid controls. In SCH women treated with LT4, the prevalence of these outcomes was comparable with that in the euthyroid women even when treatment was initiated later during pregnancy. However, considering the retrospective nature of our study and some methodological issues, our findings should be confirmed in large prospective studies.

## Data Availability

The datasets used and/or analysed during the current study available from the corresponding author on reasonable request.

## Abbreviations

ART: Assisted reproductive technology
ATA-GL: American thyroid association guidelines
BMI: Body mass index
CI: Confidence interval
GDM: Gestational diabetes mellitus
hCG: Human chorionic gonadotropin
IDA: Iron deficient anaemia
LT4: Levothyroxine
OGTT: Oral glucose tolerance test
OR: Odds ratio
PE: Preeclampsia
PTB: Preterm birth
RCT's: Randomised controlled trials
SCH: Subclinical hypothyroidism
TAI: Thyroid autoimmunity
TgAb: Thyroglobulin antibodies
TH: Thyroid hormone
TPOAb: Thyroid peroxidase antibody
